# Supplementary material for: Ecotoxicological Assessment of Polluted Soils One Year after the Application of Different Soil Remediation Techniques
Source: Toxics. 2023 Mar 24;11(4):298. doi: 10.3390/toxics11040298 (PMC10143980; doi:10.3390/toxics11040298)
Supplement: Supplementary file 1 [file toxics-11-00298-s001.zip › toxics-2308749-supplementary.pdf]

toxics

Table S1. Guideline values (mg/kg) to declare a soil as potentially polluted by legislations of different countries.

Element	Spain		China [3]	Canada [4]	Portugal [5]
	Andalusia [1]	Galicia [2]			
As	36	50	15	12	11
Cd	25	2 (1)	0.2	1.4	1
Cu	595	50	35	63	62
Pb	275	100 (80)	35	70	45
Zn	10,000	300 (200)	100	200	290

The value presented is the most restrictive by land use: Andalusia—other uses (generally, agricultural), Galicia—the same (in brackets ecosystem protection value), Canada—agricultural and/or residential/parkland use, Portugal—agricultural use and China—Level I soils.

[1] Generic Reference Value (NGR) for trace elements in the Region of Andalusia. BOJA (Boletín Oficial de la Junta de Andalucía). (2015). *Decreto 18/2015, por el que se aprueba el reglamento que regula el régimen aplicable a los suelos contaminados*. BOJA, 38, 28-64. http://www.juntadeandalucia.es/medioambiente/web/2012_provisional/2015/reglamento_suelos_contaminados.pdf

[2] Generic Reference Value (NXR) for contaminants in the Region of Galicia. DOG (Diario Oficial de Galicia). (2009). *Decreto 60/2009, do 26 de febreiro, sobre solos potencialmente contaminados e procedemento para a declaración de solos contaminados*. DOG, 57, 5920-5936. https://www.lex.gal/galilex/4384

[3] Environmental quality standards for soils (HM) in China. MEEPRC (Ministry of Ecology and Environment of the People's Republic of China). (2018). *Soil Environmental Quality Risk. Control Standard for Soil Contamination of Agricultural Land (GB 15618–2018)*. https://www.fao.org/faolex/results/details/es/c/LEX-FAOC136767/

[4] Canadian Soil Quality Guidelines. CCME (Canada Council of Ministers of the Environment). (2007). *Canadian Soil Quality Guidelines for the Protection of Environmental and Human Health: Summary Tables* (Updated September, 2007). https://support.esdat.net/Environmental%20Standards/canada/soil/rev_soil_summary_tbl_7.0_e.pdf

[5] Reference values for main soil pollutants in Portugal. APA (Agência Portuguesa do Ambiente). (2019). *Solos Contaminados - Guia Técnico - Valores de referência para solo. Revisão 3 - Setembro de 2022*. Agência Portuguesa do Ambiente. https://sniambgeoviewer.apambiente.pt/GeoDocs/geoportaldocs/AtQualSolos/Guia_Tecnico_Valores%20de%20Referencia_2019_01.pdf
